# Fractional Flow Reserve (FFR) Estimation from OCT-Based CFD Simulations: Role of Side Branches

**DOI:** 10.3390/app12115573

**Published:** 2022-05-30

**Authors:** Peshala T. Gamage, Pengfei Dong, Juhwan Lee, Yazan Gharaibeh, Vladislav N. Zimin, Hiram G. Bezerra, David L. Wilson, Linxia Gu

**Affiliations:** 1 Department of Biomedical and Chemical Engineering and Sciences, Florida Institute of Technology, Melbourne, FL 32901, USA; 2 Department of Biomedical Engineering, Case Western Reserve University, Cleveland, OH 44106, USA; 3 Department of Biomedical Engineering, The Hashemite University, Zarqa 13133, Jordan; 4 Cardiovascular Imaging Core Laboratory, Harrington Heart & Vascular Institute, University Hospitals Cleveland Medical Center, Cleveland, OH 44106, USA; 5 Interventional Cardiology Center, Heart and Vascular Institute, The University of South Florida, Tampa, FL 33606, USA

**Keywords:** fractional flow reserve (FFR), optical coherence tomography (OCT), computational fluid dynamics (CFD), hemodynamics

## Abstract

The computational fluid dynamic method has been widely used to quantify the hemodynamic alterations in a diseased artery and investigate surgery outcomes. The artery model reconstructed based on optical coherence tomography (OCT) images generally does not include the side branches. However, the side branches may significantly affect the hemodynamic assessment in a clinical setting, i.e., the fractional flow reserve (FFR), defined as the ratio of mean distal coronary pressure to mean aortic pressure. In this work, the effect of the side branches on FFR estimation was inspected with both idealized and optical coherence tomography (OCT)-reconstructed coronary artery models. The electrical analogy of blood flow was further used to understand the impact of the side branches (diameter and location) on FFR estimation. Results have shown that the side branches decrease the total resistance of the vessel tree, resulting in a higher inlet flowrate. The side branches located at the downstream of the stenosis led to a lower FFR value, while the ones at the upstream had a minimal impact on the FFR estimation. Side branches with a diameter larger than one third of the main vessel diameter are suggested to be considered for a proper FFR estimation. The findings in this study could be extended to other coronary artery imaging modalities and facilitate treatment planning.

## 1. Introduction

Pressure wire-based fractional flow reserve (FFR) has been widely used to evaluate the functional significance of a stenosis in epicardial coronary arteries, which is defined as the ratio between the mean distal coronary pressure of the stenosis (Pd) to the mean aortic pressure (Pa) during maximal hyperemia [1]. FFR-guided percutaneous coronary intervention (PCI) has shown more favorable outcomes compared with angiography-guided PCI [2]. However, hyperemic FFR is associated with additional time, cost, and more importantly, unpredictable systemic blood pressure changes and vasodilation side effects causing chest discomfort [3]. Moreover, invasive FFR shows methodological deficiencies in assessing serial lesions and early post-stented acute coronary syndrome culprit lesions. Image-derived FFR estimation is more convenient and highly promising [4–8]. Precise model reconstruction of vessel geometry is paramount in image-derived methodology, as the ambiguity in the lumen diameter has proven to have the largest impact on FFR computation [9].

Intravascular optical coherence tomography (OCT) provides much superior resolution compared to other imaging modalities [10] and has proven to deliver accurate lumen measurements with good reproducibility [11]. Several studies have employed OCT-derived vessel geometries to compute FFR by application of computational fluid dynamics (CFD) [12] or simplified fluid dynamics equations [5,13] and delivered reasonable accuracies (~85–87%) in classifying the vessels conferring to the diagnostic threshold (FFR ≤ 0.8). However, the quantitative agreement against the measured FFR was limited (with correlation coefficients ~0.7 and SD of Bland–Altman plots ~0.05–0.09), which can lead to difficulties in clinical decision making for vessels with intermediate FFR values. As OCT pullback apparently does not include the side branches’ geometry, the effect of the side branches is essentially ignored [12,13], or to a certain degree [5] in most of these studies. The study [5] assumed a varied flowrate along the vessel (derived proportionally to the reference lumen area) to indirectly account for the side branch flow and suggested an insignificant improvement in FFR prediction. Another study [14] combined CFD modeling of OCT-reconstructed vessel segments with a lump parameter model (LPM), which includes the effect of side branch flow and reported a better correlation coefficient (0.82) with FFR measurements. However, in these studies, the comprehensive analysis of the influence of the side branches on the FFR estimation is still lacking.

This study aims to inspect the influence of side branches on FFR estimation using OCT-reconstructed vessel models. First, the influence of the side branches on FFR was systematically studied with three idealized artery models and corresponding circuit models (analogous to fluid domain). The mechanistic understandings from the idealized models were further validated with two representative patient-specific models against the clinical FFR measurements. The findings of this study can help to enhance the accuracy of FFR estimation based on OCT images.

## 2. Material and Methods

Three idealized coronary artery models were constructed to quantify the effect of a side branch on FFR estimation with CFD simulations. The observations were compared with simplified electrical circuit models, which were analogous to the fluid domains. Then, FFR estimations from two patient-specific artery models reconstructed from OCT images were validated against the clinical FFR measurements.

### 2.1. Idealized Stenosed Coronary Artery Models

**Figure 1.**
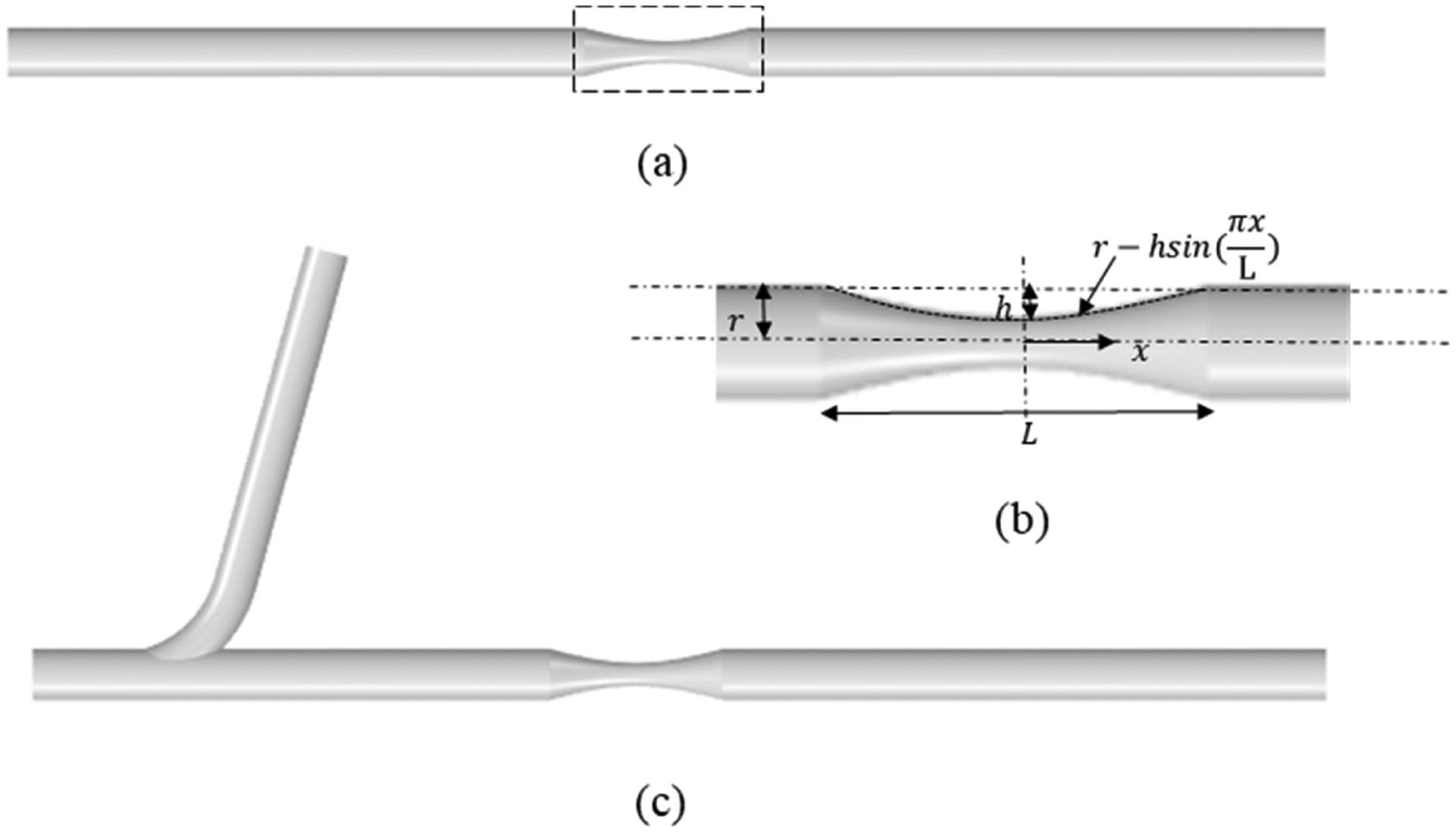
Geometric representation of the idealized stenosed artery model: (**a**) Model without side branch. (**b**) Profile of the stenosis. (**c**) Model with side branch.

Three idealized artery models—without side branch, with one side branch at the upstream of the stenosis, and with one side branch at the downstream of the stenosis—were constructed. We take the model with one side branch at the upstream as an example to illustrate the geometry of the idealized stenosed artery model (Figure 1). The length and diameter of the main vessel are 75 mm and 3 mm, respectively. The shape of the stenosis is described in Figure 1b. Stenosis is spread over a length (L) of 10 mm, and the minimum lumen area of the stenosis is 20% of the healthy lumen area. The side branch has a diameter of 2.5 mm, connects to the main vessel at an angle of 75 degrees, located 25 mm away from the center of the stenosis, at the upstream considering the flow direction.

### 2.2. IVOCT Imaging

IVOCT images were acquired with a frequency-domain ILUMIEN OCT system (St. Jude Medical Inc., St. Paul, MN, USA), which has a tunable laser light source sweeping from 1250 to 1360 nm at a frame rate of 180 fps. A 2.7-Fr OCT catheter (Dragonfly, St. Jude Medical Inc., St Paul, MN, USA) was advanced over a conventional guidewire until reaching the lesion of interest, and the catheter position was confirmed using quantitative coronary angiography (QCA). Automated pullback was then performed with contrast injection through the guiding catheter. The pullback speed was 36 mm/s with an axial resolution of 20 μm.

### 2.3. Clinical FFR Measurement

Pressure wire-based FFR was measured using 6-Fr guide catheters and a PressureWire X Guidewire (Abbott Vascular, Inc., Chicago, IL, USA). After calibration and equalization to aortic pressure, the wire was placed to at least 20 mm beyond the target lesion. Hyperemia was induced using adenosine administered by either intracoronary administration (200 μg for the left coronary artery and 100 μg for the right) or intravenous infusion at a weight-adjusted rate, equivalent to a standard dose 140 μg/kg per minute, terminated when the two minutes of measurement was completed. FFR was recorded as the ratio of distal to aortic pressure during maximal hyperemia. After recording FFR, the pressure wire was pulled back to position the sensor at the tip of the guide catheter to check the pressure drift. If the ratio of the pressure wire and guide catheter pressures differed by greater than ±0.3, FFR was re-measured after re-equalization.

### 2.4. Vessel Segmentation and Reconstruction

**Figure 2.**
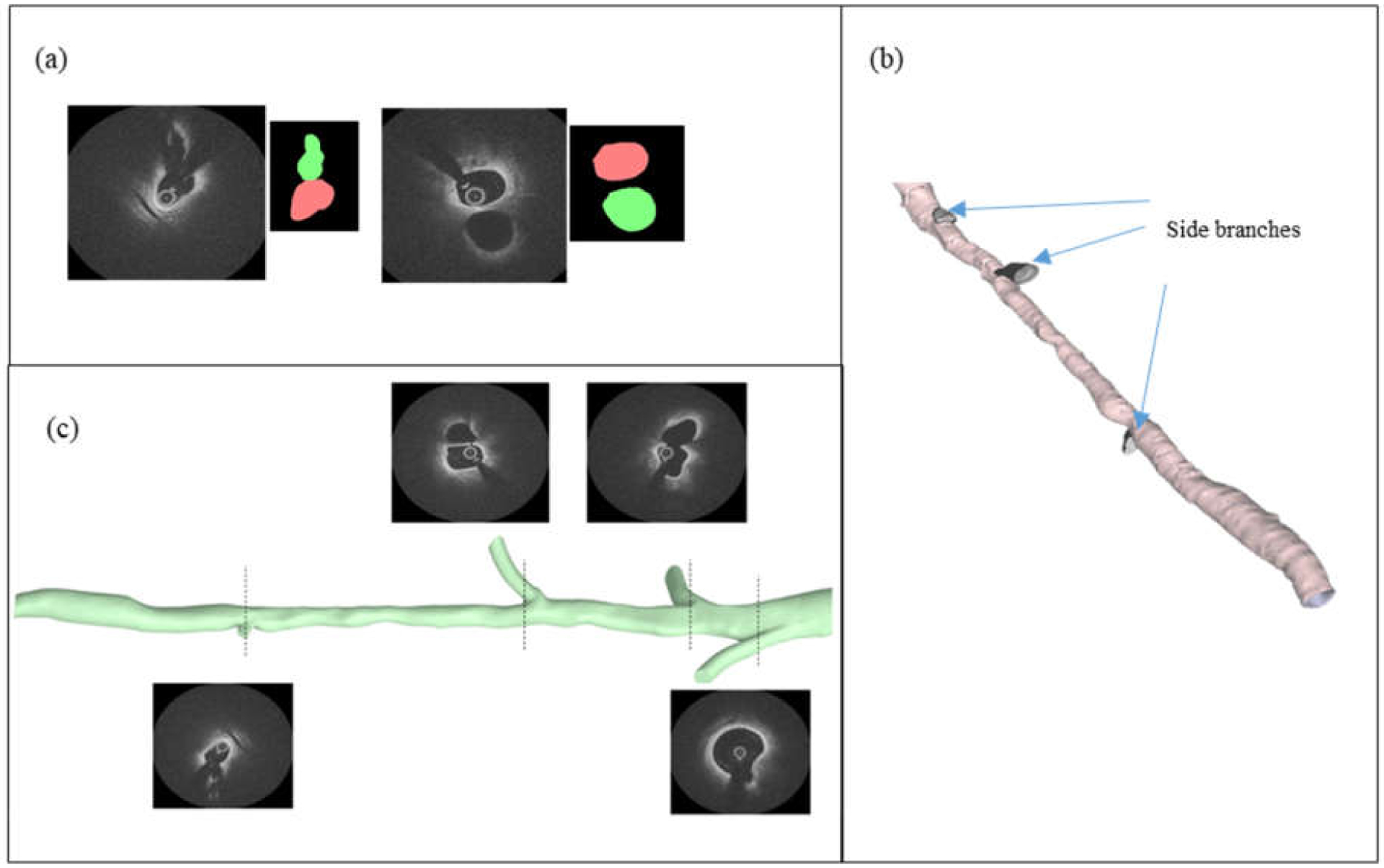
The 3D reconstruction of OCT-derived vessel tree geometry: (**a**) Segmentation of the main lumen and side branch lumen cross sections; (**b**) reconstructed geometry of the main vessel and side branches separately; (**c**) final vessel tree model generated by combining main vessel and side branches.

OCT images were used to reconstruct patient-specific artery models with and without the side branches. The 3D reconstruction process of OCT-derived artery models is shown in Figure 2. Segmentation of OCT images was performed using MATLAB image processing toolbox (2020b. The MathWorks, Inc., Natick, MA, USA). First, the lumen of the main vessel was segmented, and the extracted lumen contours from each frame were lofted along their centers to construct the 3D vessel geometry without side branches. Then, the lumen of each side branch vessel (near ostium) was manually segmented, and each side branch was separately constructed by lofting the corresponding lumen contours. This process could successfully reconstruct the segment of the side branch near its ostium, as shown in Figure 2b. The reconstructed geometries of the main vessel and the side branches in STL format were imported to ANSYS SpaceClaim (ANSYS Inc., Canonsburg, PA, USA), where they were combined to create the model with side branches. Here, the geometry was also modified by extending the side branches to improve the numerical convergence of the CFD solution.

**Figure 3.**
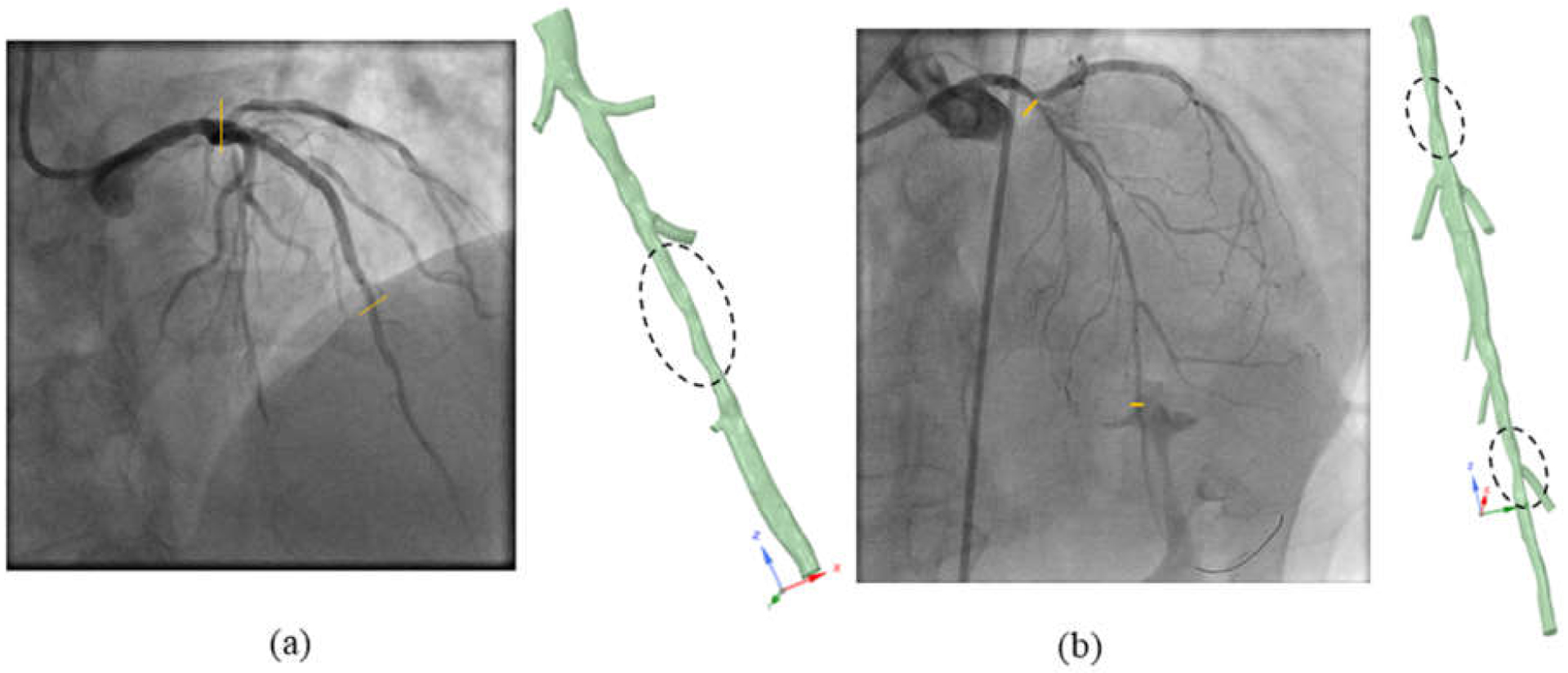
Reconstructed vessel tree models with corresponding angiography views of (**a**) patient 1 and (**b**) patient 2. Regions with significant stenosis are enclosed with dashed circles.

Figure 3 shows the 3D reconstructed vessel tree models of the two patients with corresponding angiography views. The severely stenosed regions are enclosed with dashed circles. In patient 1’s vessel tree geometry, the diffused stenosed region was located after three side branches and before a side branch with a much smaller diameter (<1 mm). In contrast to patient 1’s geometry, patient 2’s geometry had a more focal stenosed region located before the side branches and another stenosed region in the distal region of the vessel.

### 2.5. Blood Flow Modeling

The 3D reconstructed vessel models (in STL format) were imported to ANSYS ICEM CFD V.19 (ANSYS Inc., Canonsburg, PA, USA), where the fluid domain was meshed using tetrahedral elements for the computational fluid dynamic (CFD) simulation. A grid independence study was carried out to confirm the ability of the mesh to calculate the pressure with a relative error <0.01%, and the final computational mesh contained ~500,000 elements for the idealized models and ~2,000,000 elements for the patient-specific vessel models. In addition, a prism layer mesh of 4 layers with an initial thickness of 0.02 mm and a growth rate of 1.3 was employed at the wall boundaries to well resolve the flow dynamics in the boundary layer region. During meshing, a mesh smoothing algorithm was initiated to maintain a specified minimum mesh quality of 0.3, which allowed the subdivision of the elements. The final mesh had an average skewness ~0.4, where the maximum skewness was less than 0.85. These mesh quality and skewness ranges are deemed as good-quality mesh in ANSYS ICEM CFD.

The blood flow was modeled using CFD solver ANSYS CFX (ANSYS Inc., Canonsburg, PA, USA) by solving incompressible Navier–Stokes equations. A steady-state flow condition was used for calculating the FFR estimation. FFR is a time-averaged measure that is calculated over several cardiac cycles, and steady-state simulations have been used in previous work to estimate FFR [15]. Moreover, insignificant relative errors (<1%) between the transient and steady CFD solutions have been reported for FFR estimation [16]. Laminar blood flow was assumed, and a density of 1050 kgm^−3^ was used.

The fluid flow governing equations for the mass continuity and momentum conservation is given by Equation (1) and Equation (2), respectively.

(1) ∇⋅U=0

(2) ρ(U⋅∇)U=−∇P+∇⋅τ

Here, *U, ρ, P, τ*, stand for velocity vector, density, pressure, and stress tensor, respectively. Stress tensor *τ* is related to the strain rate by Equation (3),

(3) $$\tau =\mu \left(\nabla U+{\left(\nabla U\right)}^{T}-\frac{2}{3}\delta \nabla \cdot U\right)$$

where *μ*, *δ* denote the dynamic viscosity of the fluid and Kronecker delta function. To model the viscosity *μ*, the Carreau model [17] was used. The Carreau model captures the non-Newtonian, shear-thinning behavior of blood using Equation (4),

(4) $$\mu =\mu_{\infty }+\left(\mu_{0}-\mu_{\infty }\right){\left[1+{\left(\lambda \dot{S}\right)}^{2}\right]}^{\frac{n-1}{2}}$$

where *μ*_∞_ and *μ*_0_ are the viscosity value when the shear rate reaches infinity and zero, respectively. $\dot{S}$ is the shear rate. *λ* and n denote the time constant and the power law index, respectively. The following values were used in the simulations: (*μ*_∞_ = 0.0035 Pa s, *μ*_0_ = 0.25 Pa s, *λ* = 25 s, and *n*= 0.25) [18]. These values were selected by fitting the Carreau model to the viscometry measurements of Chien et al. [17] obtained for whole blood over shear rates ranging from 0.01 to 50 s^−1^.

Ansys CFX employs second-order discretization in space using a finite volume method (FVM)-based approach. The second-order upwind scheme is selected for solving the momentum equation. The software solves the coupled continuity and momentum equations using a co-located grid approach [19].

### 2.6. Boundary Conditions

A static pressure and a zero gradient boundary conditions were specified at the inlet, which are described by Equations (5) and (6).

(5) $$P=P_{in}$$

(6) $$\frac{dU}{dn}=0$$

For the idealized models, *P*_*in*_ was defined as 90 mm Hg. For the patient-specific models, the pressure was measured using the guide catheter. In Equation (6), *n* denotes the normal vector at the boundary. The artery wall was considered as stationary, and a zero-wall velocity boundary condition was imposed at the artery wall.

(7) $$U_{\text{wall}}=0$$

At each outlet, a resistance-based boundary condition was imposed to mimic the coronary microvascular resistance (CMVR) downstream from each outlet. Such a boundary condition computes the outlet pressure (*P*_*out*_) based on the flowrate (*Q*_*out*_) and the defined resistance (*R*_*out*_).

(8) $$P=P_{\text{out}}=Q_{\text{out}}R_{\text{out}}$$

An estimation of the appropriate resistance (*R*_*out*_) value at each outlet is required for an accurate computation of FFR. However, the direct measurement of the resistance of the microvascular bed at each outlet is not available and difficult to measure. Hence, to estimate the CMVR at each outlet, the structured tree model approach suggested by Olufsen et al. [20] was used. This approach has been previously employed by several studies to simulate coronary blood flow dynamics and FFR [21,22] and has shown agreement with corresponding perfusion resistance measurements [22]. This model suggests an asymmetric, fractal-like vascular tree structure based on morphological laws of vascular branching. The root of the vascular tree originates from an outlet and bifurcates into daughter vessels with diameters scaled by factors of *α* and *β*. Each branch length is derived from a length/diameter ratio (*γ*). The branching is terminated when a vessel diameter is less than a minimum diameter (*d*_*min*_), which represents the arteriolar diameter of the vascular bed [20].

For a branch segment of diameter (*d*_*i*_), the corresponding resistance (*R*_*i*_) is derived as [23],

(9) $$R_{i}=\frac{128\mu \gamma }{\pi d_{i}^{3}}$$

In addition, considering the parallel branching structure, the resistance (*R*) at any bifurcation is calculated as,

(10) $$\frac{1}{R}=\frac{1}{R_{1}}+\frac{1}{R_{2}}$$

where the resistances of the daughter vessels are denoted by *R*_1_ and *R*_2_. Subsequently, considering Ohm’s law and treating each branch as circuit elements, the total resistance of the vascular tree can be calculated. In the current study, the following parameters were used: (*α* = 0.9, *β* = 0.5, *γ* = 25 [24], *D*_*min*_ = 50 μm [20]). To simulate the hyperemic conditions, the calculated resistance is multiplied by a factor of 0.24, which corresponds to the experimental observation with intravenous administration of adenosine 140 μg/kg/min [25].

For the patient-specific models, the resistances at each side branch were determined using the structured tree approach, while the resistance at the distal end of the main vessel was determined to match the pressure wire measurement. The same distal resistance value was imposed when the model was considered without side branches.

## 3. Results and Discussion

### 3.1. FFR and Flow Distribution in Idealized Artery Models

CFD simulations were performed in three idealized artery models, namely, model 1 (without side branch), model 2 (with one side branch located at the upstream of the stenosis), and model 3 (with one side branch located at the downstream of the stenosis).

**Figure 4.**
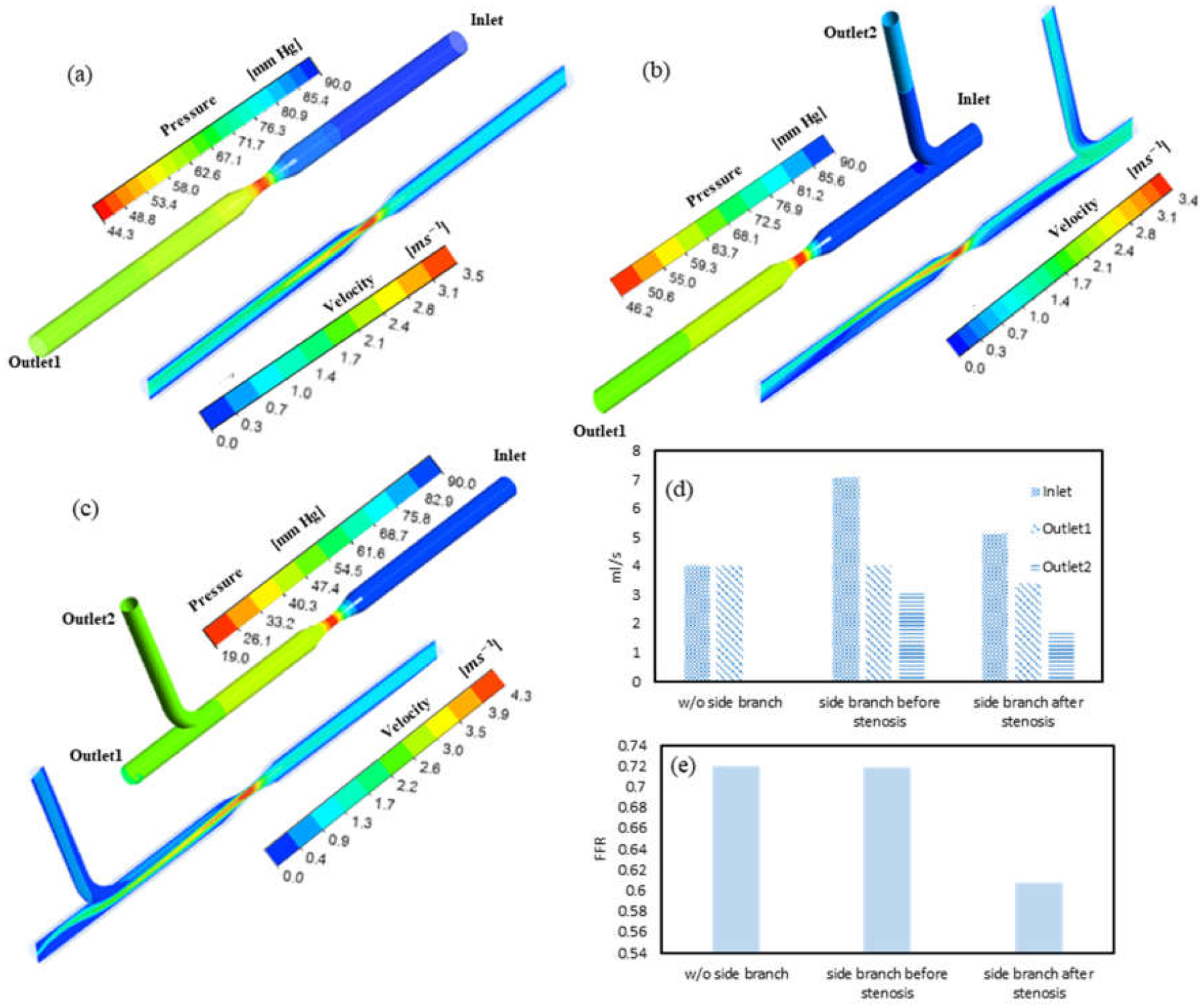
CFD-derived pressure and velocity distributions in an idealized artery model: (**a**) model 1: without side branch; (**b**) model 2: with side branch located at the upstream; (**c**) model 3: with a side branch located at the downstream. Comparison of (**d**) flowrate into side branches and (**e**) CFD-derived FFR between the three models.

The pressure and velocity distributions in the three idealized artery models are shown in Figure 4a–c. A significant pressure drop was observed as the flow passed through the stenosis. The lowest pressure was seen at the stenosis region due to a high velocity at the narrow section. As the flow traveled further downstream, the pressure slightly increased due to pressure recovery with velocity reduction. A more axial symmetric velocity distribution was observed in model 1 (without side branches) compared with the other two models (with a side branch). The blood flow was redistributed to maintain the same inlet aortic pressure, as depicted in Figure 4d. Models 2 and 3 showed a higher inlet flowrate than model 1. This indicated that the inclusion of the side branch requires more blood flow intake. Models 1 and 2 had a similar outlet flowrate from the main vessel (outlet1), i.e., <0.01% difference. It suggested a minimal alteration in the flowrate through the stenosis when the side branch is located before the stenosis region. In contrast, the outlet flowrate of the main vessel in model 3 was 15% lower than the other models. It should be noted that the flowrate through the stenosis was the same as the inlet in model 3, and as the outlet1 in the other models. This means that blood flow through the stenosis in model 3 (inlet) was 27% higher than the other models (outlet1).

The alterations in the velocity distributions caused by side branches can lead to changes in local hemodynamics parameters, such as wall shear stress (WSS), and further induce remodeling of the vessel wall. Previous studies [26,27] have revealed the importance of the inclusion of side branches when assessing the risk of atherosclerosis development in relation to WSS distribution.

FFR was determined as the ratio between the outlet pressure and the inlet pressure of the main vessel, as shown in Figure 4e. The estimated FFR of model 2 was slightly less than model 1 (with a difference of 0.002). This insignificant change in FFR is also in accordance with the similar flowrate observed through the stenosed region of these models. The estimated FFR in model 3 was significantly smaller, which was 15.5% lower compared to the other two models, suggesting that the side branch located at the downstream has a significant impact on FFR estimation.

**Figure 5.**
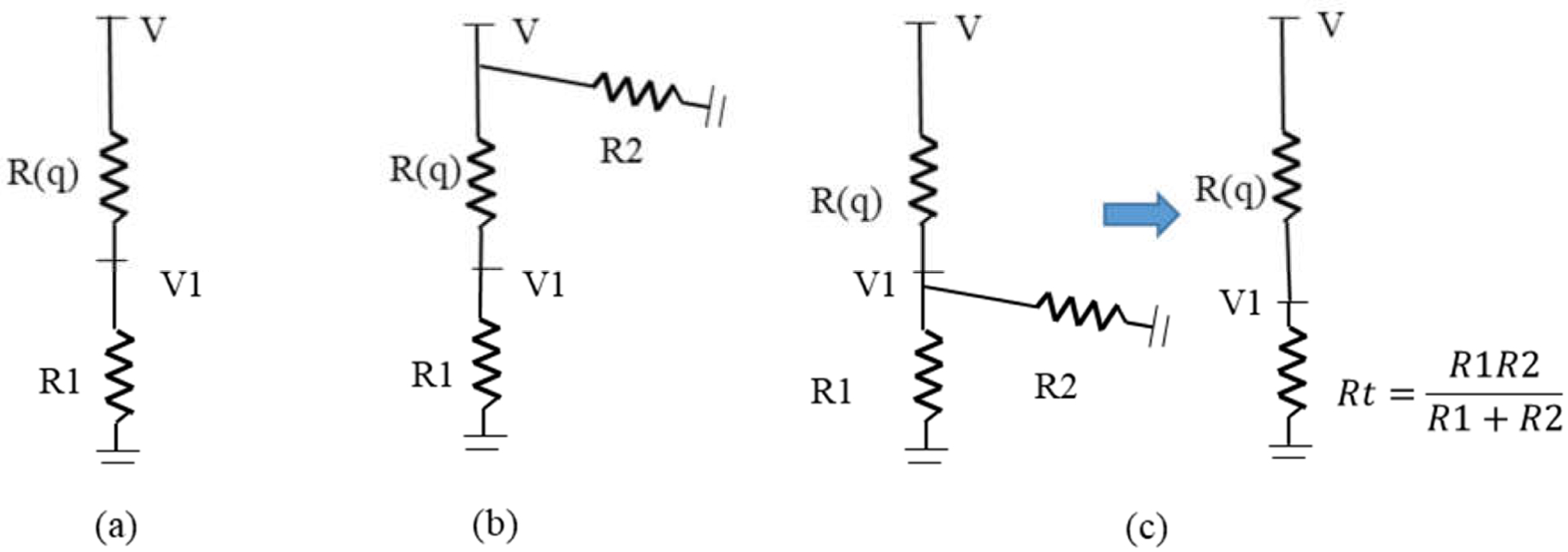
Simplified electrical circuit representation of the idealized models: (**a**) model 1: without side branch; (**b**) model 2: with side branch before the stenosis; (**c**) model 3: with side branch after the stenosis.

The influence of the side branches on the FFR value can be further illustrated with electrical circuit models (Figure 5). Here, the variables, in terms of voltage and resistance, are analogous to the pressure and flow resistance in the fluid domain, respectively. V and V1 denote the voltage (analogous to the pressure) at the main vessel inlet and outlet, respectively. R1 and R2 denote the distal microvascular resistances of the main vessel and side branch, respectively. R(q) denotes the flow resistance induced by the stenosis region. Flow resistances of the healthy segments of the arteries are assumed to be negligible compared to the stenosis region and distal microvascular resistances.

The pressure drop (*dP*) across the stenosis can be described by a nonlinear function of the flowrate (*q*) as,

(11) $$dP=C_{1}q+C_{2}q^{2}$$

where the first term and the second term of the Equation (11) represent the viscous pressure drop and expansion pressure drop across the stenosis, respectively. The parameters *C*_1_ and *C*_2_ are constants that depend on the dimensions of the stenosis, which will significantly increase along with stenosis severity, further leading to a large pressure drop. Based on Equation (11), the flow resistance through the stenosis can be derived as,

(12) $$R(q)=\frac{dP}{q}=C_{1}+C_{2}q$$

For the side branch located at the upstream of the stenosis, the resistance *R2* is connected in a parallel configuration to the resistance in the main vessel and does not affect the FFR estimation. The FFR in both model 1 and model 2 can be calculated based on *R1* and *R(q)*:

(13) $$FFR=\frac{V1}{V}=\frac{R1}{R1+R(q)}$$

As the flow in the main vessel is governed by the inlet voltage (*V)* and distal resistance (*R*1), both models have an equal flowrate in the main vessel and an equal *R*(*q*). Hence, both models lead to the same FFR. Furthermore, in these models, the flow into the side branch is determined by the inlet voltage *V* and *R2*, which are independent of *R*(*q*) and *R*1. These derivations agree well with the CFD-derived results for model 1 and model 2, where insignificant deviations in FFR and flowrate in the main vessel (outlet1) are observed.

For model 3, when the side branch is located at the downstream after the stenosis, the distal resistances *R1* and *R2* are connected in parallel, which can be represented as a total resistance (*Rt*) denoted as,

(14) $$Rt=\frac{R1R2}{R1+R2}$$

Consequently, the FFR can be calculated by Equation (15).

(15) $$FFR=\frac{V1}{V}=\frac{Rt}{R(q)+Rt}=\frac{R1}{R(q)\cdot \left(\frac{R1}{R2}+1\right)+R1}$$

In comparison to Equation (13), the term $\frac{R1}{R2}$ in Equation (15) contributes to a decrease in the FFR value. According to Murray’s law of flow distribution, the distal resistance of an artery with a diameter (*d*) is assumed to be proportional to *d*^−3^ [28]. Hence, as the main vessel has a larger diameter, the term $\frac{R1}{R2}$ is usually less than unity. In addition, $\frac{R1}{R2}$ decreases as the diameter of the side branch decreases and becomes negligible (<4%) if the side branch diameter is less than one third of the main vessel diameter. Moreover, it should be noted that the term *R*(*q*) depends on the flowrate, and for model 3, the flowrate across the stenosis was higher than the other models, as shown in Figure 4d. Hence, with the increase in flowrate across the stenosed region, the increased *R*(*q*) contributes to further decrease the FFR. The FFR and flow distribution results from the CFD simulations further validate these observations. Overall, these results suggested that the impact of side branch flow on FFR estimation is more critical when the stenosed region is located before side branches.

### 3.2. FFR Analysis in OCT-Reconstructed Vessel Models

**Figure 6.**
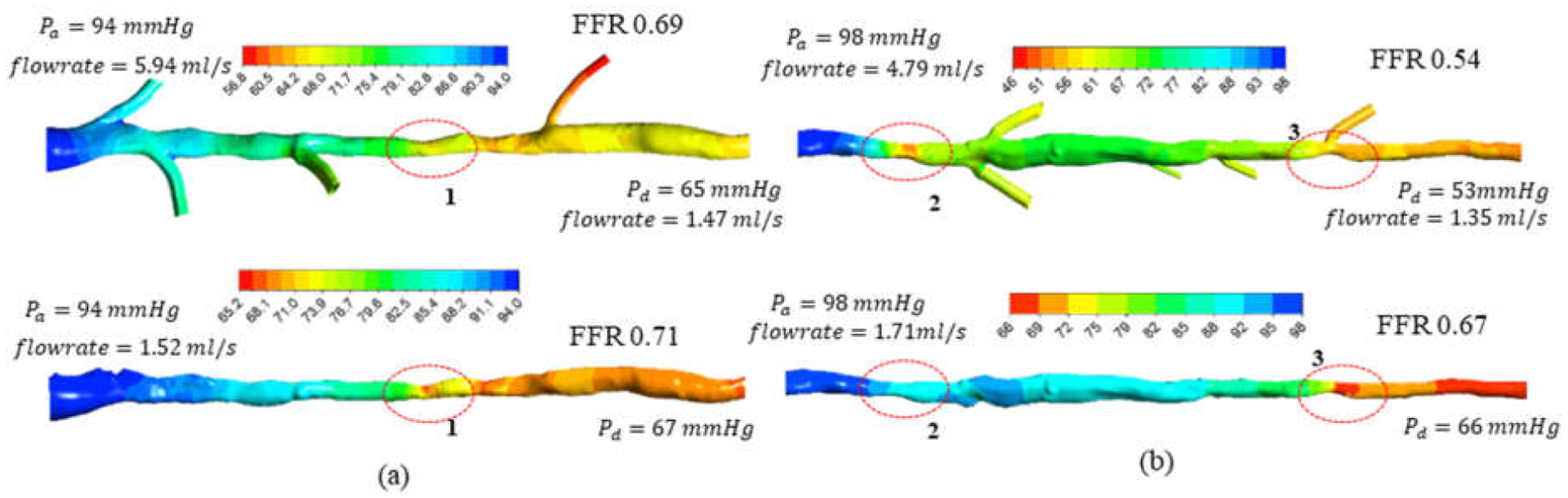
CFD-derived pressure distribution of OCT-derived vessel tree models with (top) and without (bottom) side branches for (**a**) patient 1 and (**b**) patient 2. Severely stenosed regions are enclosed with dashed lines.

The effects of the side branches on blood hemodynamics and FFR estimation were further investigated in the OCT-based vessel models from two patients (Figure 6).

In patient 1, the side branches at the upstream of the stenosis did not contribute to a significant difference in FFR estimation. In line with the observations from idealized model 2, as most of the side branches are located at the upstream of the stenosed region (denoted by region 1 in Figure 6a), the flow into these branches is likely to cause a minimum effect on the FFR estimation. The flow into the side branch located at the downstream of the stenosed region is likely to have more influence on the FFR. However, due to the smaller diameter (<1 mm) of this branch compared to the diameter (~3 mm) of the main vessel, the effect on FFR is not significant.

For patient 2, the FFR value decreased by 13% when the side branch flow was considered. This difference was more significant than the one for patient 1, which was 2%. There was a higher pressure drop for the stenosis region with side branches located at its downstream (region 2 in Figure 6b) compared with the one without side branches. As the side branches of patient 2 are located at the downstream of the stenosis (denoted by region 2 in Figure 6b), the pressure distribution in patient 2 is more likely to follow the trends of idealized model 3. As depicted using the idealized circuit model 3 (in Section 3.2 using Equation (15)), stenosis located before side branches is subjected to a higher flowrate, leading to an increased flow resistance *R*(*q*) (and pressure drop) across the stenosis. Moreover, the existence of multiple side branches after the stenosis will result in increasing the distal resistance ratio (*R*1/*R*2) between the main vessel and side branches in Equation (15), further contributing to a decrement in FFR compared to a model without side branches.

**Figure 7.**
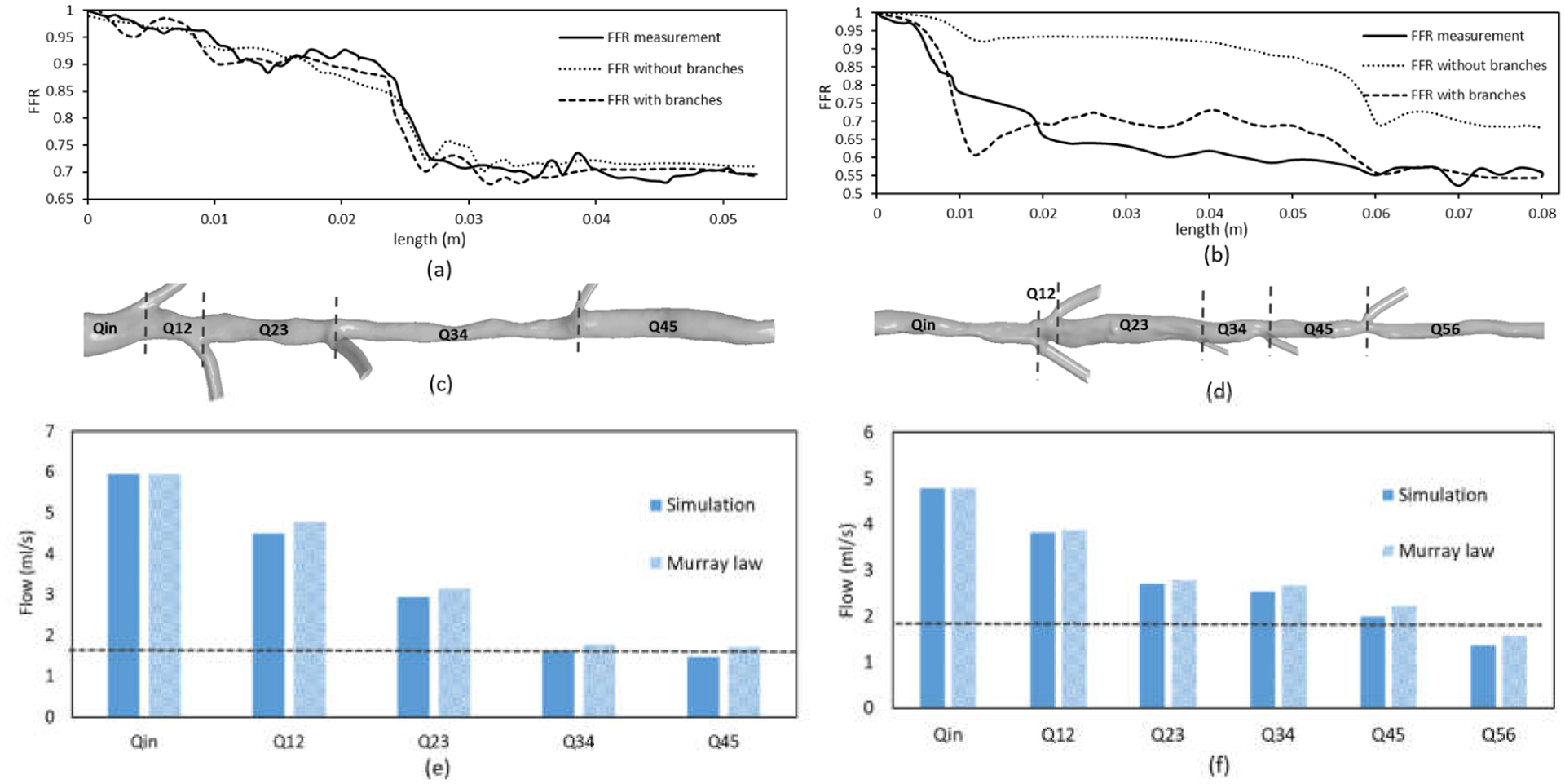
FFR variation in the main vessel compared with guidewire measurements for (**a**) patient 1 and (**b**) patient 2. Flow across the different sections (denoted in (**c**,**d**)) of the main vessel of (**e**) patient 1 and (**f**) patient 2 compared with the flow distribution derived from Murray’s law. The flowrates in the models without side branches are indicated with dashed horizontal lines in (**e**,**f**).

Overall, the FFR (or pressure drop) differences between the models with and without side branches relates to the flowrate differences in the main vessel. When side branches are considered, due to the decreased total resistance, the vessel tree intakes a much higher flowrate, and the flow along the main vessel varies as the flow leaks to the side branches. In contrast, the model without side branches intakes a lower flowrate that remains constant along the vessel. The variations of FFR and flowrate along the main vessel for patients 1 and 2 are illustrated in Figure 7. The simulated FFR variation was compared against the measurements derived from FFR pressure wire pullbacks. For patient 1, the simulated FFR variation in the main vessel with or without the side branches showed a good agreement with the measurements. Here, a significant drop in FFR was seen across the severely stenosed section (denoted by region 1 in Figure 6a). The flowrate across this section (Q34 in Figure 7c) was similar in both models with and without the side branches.

For patient 2, the FFR variation in the model with side branches showed a better agreement with FFR measurements than the one without side branches. The model neglecting the side branches at the downstream of the stenosis considerably underestimated the drop in FFR (stenosis region between Qin and Q12 in Figure 6b). Moreover, the flowrate across this region (Qin in Figure 7d) was significantly lower in comparison to the model with side branches.

The simulated flowrate variation in the main vessel was also compared with flowrate variation derived using Murray’s flow distribution law. Murray’s law assumes the flow is proportional to the third power of the vessel diameter (Q~d^3^), which is derived on the basis of adaptive mechanisms whereby blood vessels remodel to maintain homeostasis according to the shear stress levels on the endothelial surface [29]. This remodeling process is empirically validated [30] and found to be completed within a few weeks [31], and has even been observed in atherosclerotic vessels [32]. The flow distribution derived using Murray’s law showed good agreement with the simulated results, where the simulated flowrates were slightly lower (on average by ~7%). This result indicates the applicability of morphometric flow rules to modify the flowrate in the main vessel accounting for the side branch flow for a more accurate FFR estimation.

One study has analyzed the impact of side branches on FFR estimation using a computational model derived from angiography images [33]. A linearly distributed, simplified flow leakage from the main vessel was adopted in that study. A significant decrease in the inlet flowrate was reported when the side branch flow was ignored, which agrees with our findings. The same study reported relatively lower FFR values when the side branches were ignored but concluded an insignificant effect on FFR due to side branch flow. This is different from our findings; this is mainly due to the different generic distal resistance values used for the models with and without flow leakage, tuned separately to match the measured FFR values. In contrast, the current study imposed the same distal resistance at the main vessel exit in models with and without side branches for comparison purposes. Moreover, analytical findings in our study suggested that the effect of side branch flow is case-specific depending on the location of the stenosis in the main vessel.

Another study [27] has investigated the impact of side branches on hemodynamic indices using vessel tree models reconstructed by the fusion of OCT and angiography images and reported a significantly higher distal coronary pressure to aortic pressure ratio (*P*_*d*_/*P*_*a*_) in vessel tree models compared to a single lumen model (without branches). However, that study imposed the same inlet mass flowrates in the vessel tree and single lumen model. Hence, the flowrate in the single lumen model is implicitly forced to be higher, which leads to higher pressure drops than the vessel tree model. In contrast, the more realistic distal resistance and inlet pressure boundary conditions were used in the current study to permit the flow to adjust to the model geometry based on distal resistances. This highlights the significance of choosing the appropriate boundary conditions for FFR prediction when the side branch flow is considered.

### 3.3. Implications on FFR Computation Using OCT Imaging

The FFR estimation with OCT-based computational models have usually ignored the presence of the side branches [12,13], which may cause a deviation compared with the FFR measurement. Some studies have shown an improvement in FFR prediction by implicitly accounting for the side branch flow [5,14]. This work suggests that the side branch at the downstream to the stenosis is necessary to consider for a precise FFR estimation with CFD simulation. In contrast to other imaging modalities, such as computed tomography (CT) angiography and 3D rotational angiography, OCT images cannot directly capture the side branches, as the images are acquired through a catheter guided along the main vessel. Yet, these images contain the information of side branch ostia, which can be used to approximate the geometry of the vessel tree structure. The accurate segmentation of the side branch ostia from OCT imaging is critical for estimating the side branch flow. While manual segmentation can be time-consuming and labor-intensive, automatic side branch ostium segmentation algorithms for OCT imaging are also available [34]. Moreover, more accurate 3D vessel geometry can be reconstructed by combining OCT images with angiographic views [27].

Varied hyperemic boundary conditions in different patients brings more uncertainty in FFR predictions [35]. Previous studies have suggested different methods of non-invasively estimating the patient-specific hyperemic boundary conditions. One popular method of estimating the patient-specific boundary conditions is based on determining the myocardial mass and vessel diameters [36]. This method employs allomeric laws to determine coronary flow from myocardial mass and subsequently estimates the distal resistances based on vessel diameters as governed by Murray’s law. However, CT imaging is required for calculating the myocardial mass and is thus not appropriate for the OCT-based method. One previous study [14] suggested a similar method of estimating the distal resistances based on the length of coronary arteries extracted from angiographic views and used to predict FFR in vessel tree models reconstructed using OCT imaging. Another study [37] estimated the patient-specific hyperemic flow intake through allometric relations and implemented a non-linear boundary condition to estimate the distal resistances by allowing the modification of Murray’s law flow distribution in the diseased artery tree. The same study [37] estimated the FFR in vessel tree models reconstructed from intravascular ultrasound (IVUS) imaging with the side branches manually segmented, which can also be implemented with OCT imaging. Moreover, more simple approaches have been used to specify the flow fraction boundary conditions at the outlets according to morphometric flow rules, assuming the deviations in flow distribution (from the healthy state) are insignificant in the diseased arteries [15,38], which is also seen in our study. The use of morphometric flow rules to correct the flow in the main vessel can serve as a reasonable approximation, which can be combined with previously suggested equation-based FFR estimation methods [5,13] to improve their performance.

## 4. Limitations

The curvature of the arteries is not considered in the current geometry reconstruction process, which may deviate the FFR computations. However, previous studies reported minor effects on FFR caused by the curvature of the coronary arteries [39,40]. For more accurate computations, 3D curved vessel geometry can be reconstructed by placing the OCT-segmented lumen cross sections in the 3D curvature of the artery derived from different angiographic views [41,42]. The side vessels are manually segmented and connected to the main vessel, where very small branches (<0.5 mm) were ignored. The errors in segmented side branch diameters, angles, and locations may deviate the flow distribution into side branches. However, these errors are likely to cause a minimum effect on FFR computation and have less impact on the FFR comparisons in the study. More accurate segmentation of the side branches can be achieved by the fusion of OCT images and angiography views [27] or through automated segmentation methods [34]. The microvascular resistances of the side branch outlets were calculated by assuming a structured tree model [20] connected to the outlets. While these resistance values are likely to vary from the realistic patient-specific microvascular resistances, they provide a reasonable approximation for the aim of the study, exploring the effect of side branch flow on FFR. The study only incorporated two patient-specific vessel models to evaluate the conclusions derived from the idealized models. However, the qualitative findings are expected to remain the same. Analysis of FFR in more patient-specific vessels will further strengthen the findings of the study.

## 5. Conclusions

This study investigated the influence of side branches on FFR estimation. First, the influence of side branches on FFR estimation was investigated with three idealized models and further interpreted with circuit models. The understandings from the idealized models were further validated with two patient-specific models. The inclusion of side branches caused a significant increase in the inlet flowrate. The side branches at the downstream of the stenosis will significantly reduce the FFR value, while the side branches at the upstream of the stenosis have a minimal effect on the FFR estimation. The side branches with a smaller diameter have less influence on the FFR estimation. The findings of this study could enhance the understanding of the relationship between pressure, the anatomy of narrowing vessels, and the anatomy of a vessel tree, which are not limited to OCT-derived vessel geometries. Accounting for the blood flow into side branches will enhance FFR estimation accuracy and likely produce a better quantitative agreement with patient-specific FFR measurements compared to the models that ignore the side branch flow.
